# Sustained clinical benefit of AAV gene therapy in severe hemophilia B

**DOI:** 10.1056/NEJMoa2414783

**Published:** 2025-06-12

**Authors:** Ulrike M. Reiss, Andrew M. Davidoff, Edward G.D. Tuddenham, Pratima Chowdary, Jenny McIntosh, Vincent Muczynski, Malo Journou, Giulia Simini, Lydia Ireland, Saira Mohamed, Anne Riddell, Arnulfo J. Pie, Andrew Hall, Alberto Quaglia, Sarah Mangles, Johnny Mahlangu, Kristina Haley, Michael Recht, Yu-Min Shen, Kathleen G. Halka, Gail Fortner, Christopher L. Morton, Zhengming Gu, Randall T. Hayden, Ellis J. Neufeld, Victoria I. Okhomina, Guolian Kang, Amit C. Nathwani

**Affiliations:** Department of Hematology, St. Jude Children's Research Hospital, Memphis, TN, USA; Department of Surgery, St. Jude Children's Research Hospital, Memphis, TN, USA; UCL Cancer Institute, London, UK; Katharine Dormandy Haemophilia and Thrombosis Unit, Royal Free Hospital, London, UK; Katharine Dormandy Haemophilia and Thrombosis Unit, Royal Free Hospital, London, UK; UCL Cancer Institute, London, UK; UCL Cancer Institute, London, UK; Basingstoke and North Hampshire Foundation Trust, UK; Katharine Dormandy Haemophilia and Thrombosis Unit, Royal Free Hospital, London, UK; Sheila Sherlock Liver Centre, Royal Free Hospital, London, UK; Basingstoke and North Hampshire Foundation Trust, UK; Department of Molecular Medicine and Haematology, Faculty of Health Sciences, University of the Witwatersrand, and Charlotte Maxeke Johannesburg Academic Hospital, Johannesburg, South Africa; The Hemostasis & Thrombosis Center, Oregon Health & Science University, Portland, Oregon; The Hemophilia Treatment Center and Pediatric Hematology-Oncology, Yale University School of Medicine, New Haven, CT, USA; Department of Internal Medicine, University of Texas Southwestern Medical Center, Dallas, TX, USA; Baylor Scott & White Healthcare, Temple Clinic, Temple, TX, USA; Department of Hematology, St. Jude Children's Research Hospital, Memphis, TN, USA; Department of Surgery, St. Jude Children's Research Hospital, Memphis, TN, USA; Department of Pathology, St. Jude Children’s Research Hospital, Memphis, TN, USA; Department of Hematology, St. Jude Children's Research Hospital, Memphis, TN, USA; Department of Biostatistics, St. Jude Children's Research Hospital, Memphis, TN, USA; UCL Cancer Institute, London, UK; Katharine Dormandy Haemophilia and Thrombosis Unit, Royal Free Hospital, London, UK

## Abstract

We report the 13-year follow-up of gene therapy in 10 adults with severe hemophilia B. A single infusion of scAAV2/8-LP1-hFIXco at one of three dose levels resulted in stable transgenic factor IX activity (mean 1.7, 2.3 and 4.8 IU/dL) leading to a median 9.7-fold (IQR, 3.7 to 21.8) reduction in bleeding rate and a 12.4-fold (IQR, 2.21 to 27.1) decrease in factor IX concentrate usage. Fifteen treatment-related adverse events occurred, primarily transient liver enzyme elevations. No cases of inhibitor, thrombosis, recurrent liver injury, or death were reported. Two malignancies were likely unrelated to the vector. These findings support the long-term promise of gene therapy for hemophilia B, demonstrating durable transgene expression with sustained clinical benefit, without late toxicity. (Funded by the Medical Research Council, U.K., and others. ClinicalTrials.gov: NCT00979238; Eudract 2018-001333-40)

Hemophilia B, an X-linked recessive bleeding disorder, arises from mutations in the *F9* gene, leading to reduced production of functional coagulation factor IX (FIX) protein. Individuals with severe hemophilia B (<1% or <1IU/dL FIX activity) suffer from frequent spontaneous bleeds, resulting in chronic arthropathy and potentially life-threatening hemorrhages.^1^ The current standard of care, lifelong factor IX concentrate prophylaxis, while improving outcomes, remains invasive, costly, and burdensome.^1^

In 2014, we reported successful gene therapy in severe hemophilia B patients (NCT00979238) following a single intravenous infusion of a self-complementary, serotype-8 pseudotyped adeno-associated virus (AAV) vector encoding the wild-type, codon-optimized factor IX gene (scAAV2/8-LP1-hFIXco).^2,3^ We demonstrated that glucocorticoids effectively suppressed immune responses against AAV-transduced hepatocytes, preserving transgenic factor IX levels. Subsequent studies by others, including those using the Padua gain-of-function factor IX variant, validated these findings, paving the way for the conditional marketing authorization of etranacogene dezaparvovec (Hemgenix) and fidanacogene elaparvovec (Beqvez) gene therapies for adults with severe hemophilia. These market approved therapies achieve higher factor IX activity levels but comparable factor IX protein levels.^4,5^

However, uncertainties remain regarding the durability of transgene expression, as the AAV transferred expression cassettes are primarily retained as episomes and subject to loss over time with division of transduced hepatocytes.^6^ Additionally, long-term safety of AAV-mediated gene transfer remains unknown and the impact of the humoral immune response to AAV requires ongoing evaluation.

Here, we present safety and efficacy data from ten men with severe hemophilia B treated with a single bolus infusion of scAAV2/8-LP1-hFIXco, followed over a median of 13 years. We demonstrate durable transgene expression providing sustained protection from spontaneous bleeding in the absence of long-term safety issues. These findings offer valuable insights for currently approved hemophilia B gene therapies.

## Methods

### Study participants

Ten men with severe hemophilia B received a single dose of scAAV2/8-LP1-hFIXco vector via peripheral vein in one of three dose cohorts (low, 2x10^11^ vector genome copies (vg) per kilogram, N=2; intermediate, 6x10^11^ vg per kilogram, N=2; or high, 2x10^12^ vg per kilogram, N=6) between March 2010 and December 2012 (Table 1) as described before and detailed in the Supplementary Appendix (Table S1).^2,3^ The scAAV2/8-LP1-hFIXco vector has been described previously.^7,8^ Safety and efficacy assessments included routine laboratory studies, factor IX activity, annualized bleeding rate (ABR), factor IX concentrate usage, and immune responses. Median follow-up, as of December 31, 2023, has been 13.0 years (range 11.1-13.8 years). A liver biopsy was performed on one participant 10 years post-gene therapy for molecular analysis under a separate protocol (Eudract 2018-001333-40).

### Trial Oversight

This study was sponsored by St. Jude Children’s Research Hospital (SJCRH). The protocol, developed jointly by the authors and sponsor, was overseen by a trial steering committee, an independent data and safety monitoring committee, and a trial management group. A confidential disclosure agreement between sponsor and study sites was maintained throughout the study. Principal investigators collected data, which were analyzed in collaboration with the statisticians at SJCRH. The authors affirm the completeness, accuracy, and protocol fidelity of the data (protocol available at NEJM.org). The manuscript was drafted by a working group including the first two and corresponding authors, then revised and approved by all authors. The trial management group approved submission.

## Results

### Long-term safety of scAAV2/8-LP1-hFIXco

Since 2010, 354 adverse events were reported (Table S2, Supplementary Appendix). No cases of factor IX inhibitor, thrombosis, recurrent transaminitis, or death were observed. Fifteen adverse events were linked to AAV gene therapy, including transient liver transaminase elevations (Grade 1-2) in 4 of 6 participants treated with the high vector dose, leading to transgene expression loss in two cases due to delayed glucocorticoid treatment.^2,3^ By month 5, alanine aminotransferase (ALT) and aspartate aminotransferase (AST) levels normalized. Liver ultrasounds and chest CT scans in compliant participants showed no ongoing liver damage, fibrosis, malignancy, or lung pathology.

Per protocol, two malignancies were reported to the regulators as serious adverse events: (1) lung adenocarcinoma in situ, detected incidentally after bullectomy for recurrent pneumothorax 5 years post-therapy in a 44-year-old with an approximately 10 pack-year smoking history over 27 years, and (2) prostate adenocarcinoma in a 74-year-old 11.6 years post-therapy. Molecular analyses and expert multidisciplinary team review suggested both were likely unrelated to gene therapy. Further details are in the Supplementary Appendix.

### Sustained clinical benefit of scAAV2/8-LP1-hFIXco

**Figure 1:**
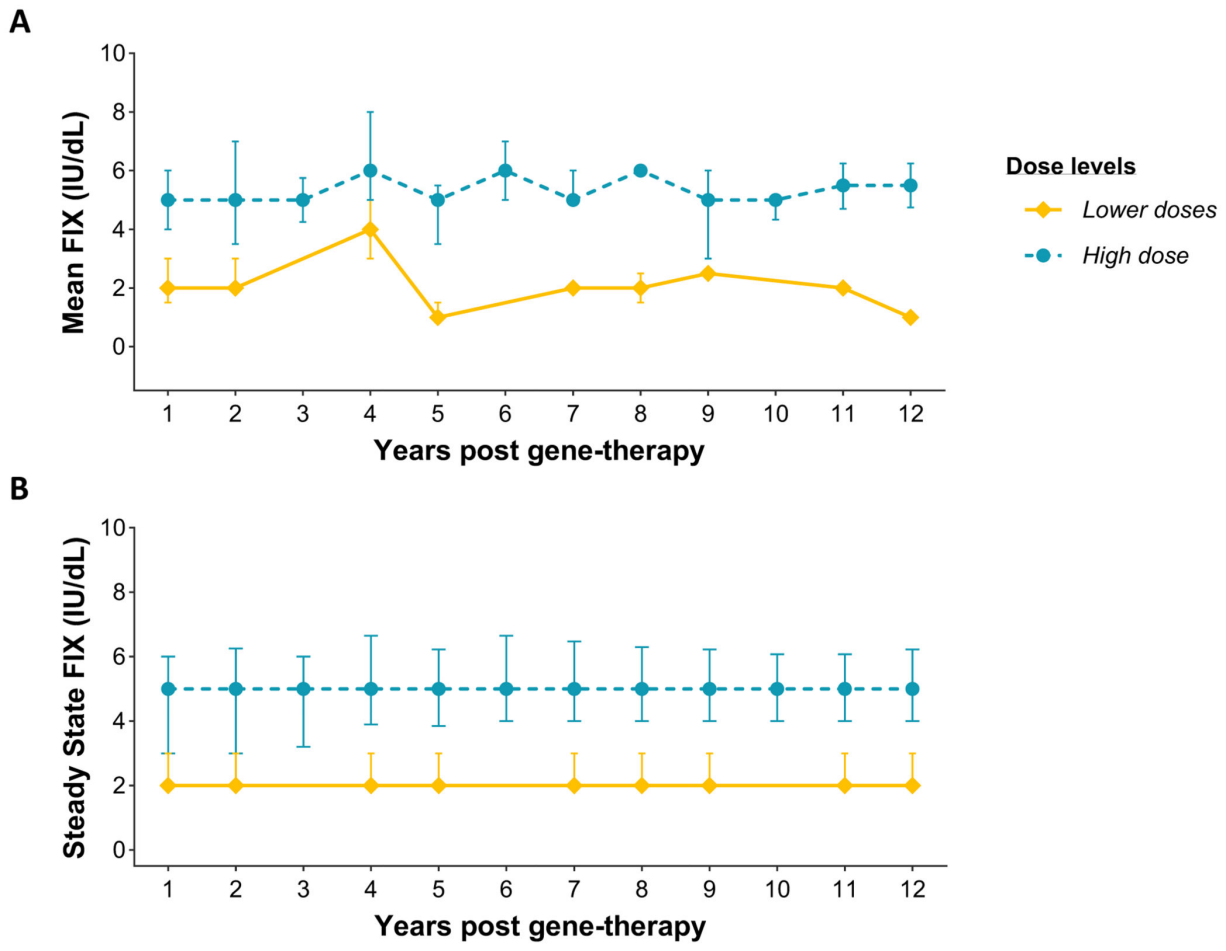
Factor IX activity levels following peripheral vein administration of scAAV2/8-LP1-hFIXco using factor IX levels uninfluenced by prophylaxis use or treatment with factor IX concentrate. Annual and steady-state FIX:C (median, interquartile range) was determined at the indicated time points using a one-stage clotting assay following administration of the high vector dose (2x10^12^ vg per kilogram of body weight, dotted blue line, N=6) compared with levels in the low (2x10^11^ vg per kilogram) and intermediate (6x10^11^ vg per kilogram) vector doses combined (N=4, solid yellow line). Annual factor IX activity **(A)** was determined for each group using the mean factor IX activity collected during the indicated time point. Steady-state FIX:C **(B)** for each group was the average of all accumulated factor IX activity levels from five months post-gene therapy up to the indicated time point. Only factor IX levels measured at least 10 days after factor IX use were included.

At a median of 3.2 years after infusion of scAAV2/8-LP1-hFIXco, all participants exhibited dose-dependent increases in factor IX coagulant activity to a mean of 1.8 ± 0.7 IU/dL, 2.5 ± 0.9 IU/dL, and 5.1 ± 1.7 IU/dL for the low, intermediate, and high-dose cohorts, respectively (mean of all levels after month 4).^2^ Over a median study period of 13 years, mean factor IX activity remained stable, with values of 1.7 ±0.3, 2.3 ±0.5, and 4.8 ±1.7 IU/dL. This resulted in a 3.3 IU/dL (high dose median: 5.18, IQR, 3.5 to 5.7; low and intermediate dose median:1.9, IQR, 1.8 to 2.1) difference in steady-state factor IX activity levels between the high-dose group and the combined low and intermediate-dose groups as of December 31, 2023 (Figure 1). Three participants with severe hemophilic arthropathy (Participants 2, 3, and 5, median number of target joints = 10) resumed factor IX prophylaxis within four years of gene therapy due to recurrent spontaneous joint bleeds. Factor IX levels of 1–3 IU/dL in these individuals measured at least three days post-infusion, proved insufficient to prevent such bleeding, highlighting the impact of joint health and other biological factors on outcomes post-gene therapy. In contrast, seven participants (Participants 1, 4, 6, 7, 8, 9, and 10) sustained factor IX levels of 2–7 IU/dL and remained off prophylaxis during long-term follow-up.

Before gene therapy, the median ABR was 14 episodes (interquartile range [IQR], 12 to 21.5) for all 10 participants, including 3 participants receiving on-demand treatment. With nearly 13 years of follow-up, the median ABR for the 10 participants was 1.5 episodes (IQR, 0.7 to 2.2) (Figure S1, Supplementary Appendix), representing a median 9.7-fold (IQR, 3.7 to 21.8) reduction in bleeding events compared to the pre-treatment period. In the 6 high-dose participants, the ABR decreased by a median of 16.4-fold (IQR, 9.7 to 31.3) to 1 bleeding episode annually (IQR, 0.4 to 2.0) compared to a median of 21 episodes (IQR, 16.2 to 27.2) pre-gene therapy. Post-hoc assessment of bleeding events showed a decline from a median of 5.26 (IQR, 3.27 to 9) at 1-year post-gene therapy to a median of 1.5 (IQR, 0.6 to 2.3) bleeds at 11 years post-gene therapy. These reductions (median fold change=3.9, IQR, 2.1 to 8.2) in ABR over a decade following infusion suggest that sustained, albeit low-level, factor IX expression may attenuate pre-existing synovitis and inflammation, potentially providing long-term protection against bleeding and mitigating further joint damage. These findings align with observations from hemophilia A gene therapy studies, warranting further investigation.^9,10^

The median annual factor IX concentrate usage before gene therapy was 2613 IU per kilogram (IQR, 1671 to 4513). Over 13-years following gene therapy, factor IX concentrate usage decreased a median 12.4-fold (IQR, 2.21 to 27.1) to 367 IU per kilogram (IQR: 60 to 1597) at 13 years (Figure S2, Supplementary Appendix). In the high-dose group, usage dropped from 2613 IU/kg (IQR, 1627–3487) to 171 IU/kg (IQR, 60–432), a median 14.7-fold (IQR, 11.9 to 27.1) reduction.

A transjugular liver biopsy in participant 8 from the high-dose group, 10 years post-gene therapy, showed preserved lobular architecture with no necrosis, fibrosis, or dysplasia. In situ hybridization (ISH) detected hFIXco DNA in 10.3 ±3.4% of hepatocytes, while RNA in situ hybridization (RISH) revealed active transcription in 5.5 ±2.3%, indicating that just over half of the transduced hepatocytes were transcriptionally active in transgene-positive cells. No segregation between active and inactive hepatocytes was observed (Figure S3, Supplementary Appendix). This single liver biopsy provides data limited to the sampled region and may not fully reflect transgene expression across the entire liver. Nevertheless, it demonstrates persistent transgene transcription within a subset of transduced hepatocytes.

### Persistence of high titer neutralizing AAV antibodies over time

**Figure 2:**
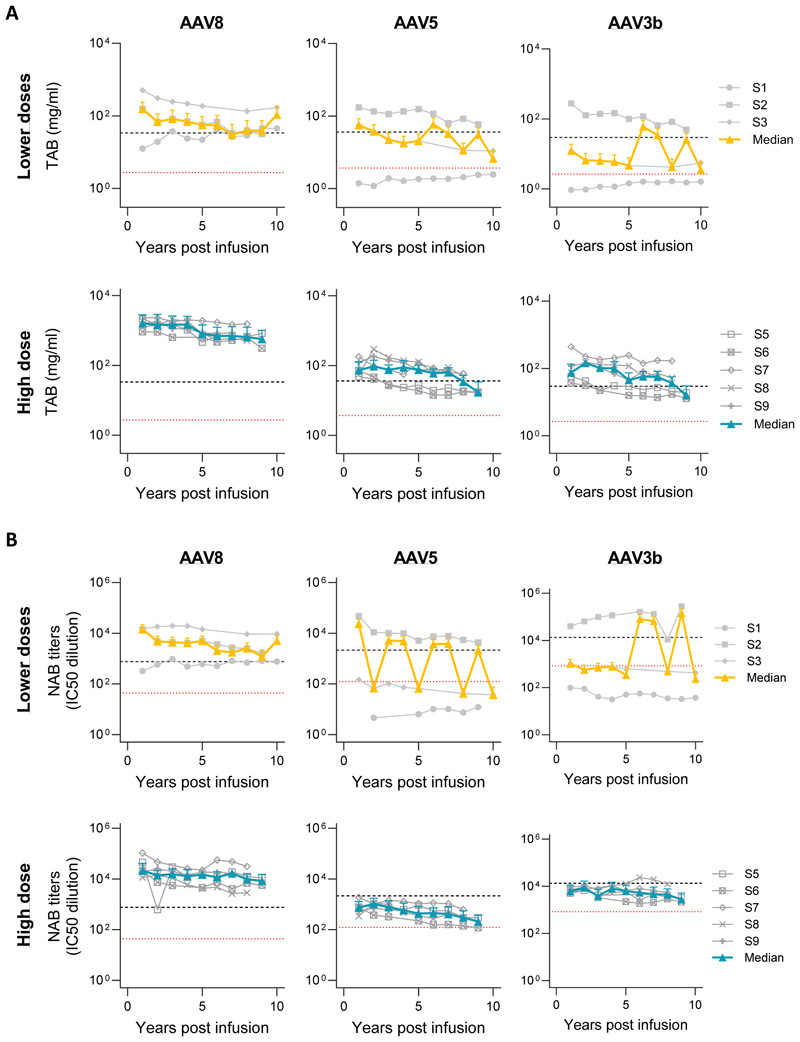
Longitudinal analysis of anti-AAV capsid antibody level following peripheral vein administration of scAAV2/8-LP1-hFIXco. Total IgG antibody levels by ELISA **(A)** or neutralizing antibody levels by transduction inhibition assay **(B)** against AAV8, AAV5 and AAV3b in serum samples collected over time after gene transfer in individual subjects treated at the low and intermediate vector dose levels combined (Lower doses) or the high dose. Shown are antibody levels over time for individual subjects (grey lines) as well as the median and interquartile range (25^th^ percentile=bottom bar, 75^th^ percentile=top bar) for the dose level (solid yellow line and "I" bars for low and intermediate dose, solid blue line and "I" bars and for high dose). For reference, the maximum (dashed black line) and median (dotted red line) AAV antibody levels observed in 38 samples from individuals presumed to have been infected with wild-type AAV are shown.

A dose-dependent increase in total IgG antibodies and neutralizing antibodies against AAV8 capsid was observed in all participants, with both measures showing a similar profile over the follow-up period (Figure 2). At one-year, neutralizing antibody levels increased a median of 3721.3-fold (IQR, 1374.7 to 6640) and remained >300-fold higher than controls (392.5-fold, control median= 43.6, IQR 5.0 to 96.5; participant median = 17100.0, IQR 13530.2 to 27125.0). By five years, neutralizing antibody levels in the high-dose cohort declined but were still >2,400-fold above baseline (median=2427.8-fold, IQR 459.1 to 3340), exceeding the predefined threshold for successful gene transfer, which is based on preclinical in-vivo experience. Cross-reactivity with AAV5 and AAV3b was dose-dependent. In murine assays, patient sera >5 years post-therapy inhibited AAV8 transduction but allowed limited AAV5 transfer, suggesting persistent NABs may hinder repeat dosing, while alternative serotypes may offer a viable option for retreatment in the future (Figure S4, Supplementary Appendix).

## Discussion

This 13-year longitudinal study provides long-term observation data in patients who had successful AAV gene therapy for severe hemophilia B. Beyond transient liver transaminase elevations, no long-term or new AAV-related adverse events were observed. Factor IX expression remained stable with seven of ten participants remaining off factor IX prophylaxis. Clinically, AAV-gene transfer resulted in an >9-fold reduction on in ABRs and factor IX concentrate usage, significantly alleviating the disease burden. These findings support the long-term safety and efficacy of AAV gene therapy for hemophilia B, offering this group of patients a promising and durable treatment option through recently licensed gene therapy products.

Two participants developed neoplastic lesions, which were reported as serious adverse events possibly related to AAV per protocol. However, subsequent molecular investigations and expert multidisciplinary review suggested these events were likely unrelated to AAV gene therapy, attributing them instead to age-related or environmental risk factors prevalent in the general population, as previously described.^4^ It is important to note, however, that long-term studies in hemophilia A dogs revealed clonal expansion of hepatocytes with AAV vector insertions near genes associated with human cancers.^11^ While no overt nodule formation or transformation was observed in these dogs, this finding highlights the importance of long-term surveillance and further investigation into the potential long-term effects of AAV-mediated gene therapy.

As reported in other studies, an asymptomatic, vector-related, rise in liver transaminases (Grade 1-2) occurred in some participants but did not result in lasting impairment of liver function.^2,3,12^ Glucocorticoids appeared to effectively manage the liver transaminases. While the precise mechanism of hepato-cellular injury remains unclear, close monitoring is crucial due to the potential for late liver enzyme elevations as well as fulminant liver failure. ^13 14, 15, 16,17^ A liver biopsy from a participant 10-years post-gene transfer demonstrated that the sustained transgenic factor IX expression and hemostatic protection in this individual was driven by a small subset of transcriptionally active hepatocytes, with no evidence of active inflammation or histological abnormalities. While integration analysis was not feasible due to lack of sufficient DNA, we hypothesize that both episomal and integrated forms of the AAV vector contribute to factor IX expression in this individual based on the current understanding of the AAV lifecycle in humans and animal models.^6,18^ This likely occurs within the context of natural hepatocyte turnover (~12 months in adults), which may contribute to the gradual loss of episomal genomes.^19,20^

Durable AAV-mediated transgene expression with concomitant clinical improvement has also been observed in other disorders, including spinal muscular atrophy and inherited retinal dystrophy^21,22^ However, decline or loss of transgene expression has been reported in both animal models and humans.^23,15^ This suggests that durability of transgene expression is complex and multifactorial, involving a dynamic interplay between the AAV genome, target disease, immunological and non-immunological factors encompassing vector design, epigenetics, as well as patient-specific considerations.^4^

Humoral immune responses to AAV have been observed across various diseases, delivery routes, and serotypes, but their long-term persistence in humans remains underexplored. In our study, high-titer neutralizing antibody against AAV8 persisted for at least 10 years post-gene therapy, with broad cross-reactivity, consistent with other reports.^24^ While neutralizing antibody levels after environmental exposure are comparable between humans and non-human primates, the response to intravenous recombinant AAV administration was magnitudes higher in humans.^25^ This suggests species-specific differences in immune responses. Although alternative serotypes may offer potential for vector re-administration, their efficacy in fully overcoming persistently high levels of cross-reactive neutralizing antibody in humans remains uncertain, suggesting that AAV-mediated gene therapy for most patients is a one-time opportunity.

In summary, this 13-year longitudinal follow-up of men with severe hemophilia B confirms the long-term safety of AAV gene therapy associated with durable factor IX expression, accompanied by lasting improvement in hemostasis and reduction in the need for factor IX prophylaxis.

## Supplementary Material

Supplement

## Figures and Tables

**Table 1:** Characteristics of Patients at Screening and after Gene Transfer

Participants	1	2	3	4	5	6	7	8	9	10
***Dose level (vg/kg)***	**Low (2x10^11^)**		**Intermediate (6x10^11^)**		**High (2x10^12^)**					
***Age at study entry (years)***	31	64	43	29	32	27	22	38	44	33
***FIX Mutation***	31280 G>A E387K	2bp del. Frame shift	30097 G>T W215C	31290 G>A A309T	20518 C>T R180W	-52 del C	3bp del Frame shift	1277 C>T T426I	698 C>A A233D	385 G>T G129X
***CRM status***	Pos	Neg	Pos	Pos	Pos	Neg	Neg	Pos	Pos	Neg
***Prophylaxis before gene therapy***	2X/week	2X/week	2X/week	1X/week	2X/week	3X/week	1X/week	On- demand	On- demand	On- demand
***No of target joints***	4	10	6	3	10	2	2	4	5	3
***Infection markers***										
Hepatitis B surface antigen	Neg	Neg	Neg	Neg	Neg	Neg	Neg	Neg	Neg	Neg
Hepatitis B surface antibody	Pos	Pos	Pos	Pos	Pos	Pos	Neg	Pos	Pos	Pos
HIV antibody	Neg	Neg	Neg	Neg	Neg	Neg	Neg	Neg	Neg	Neg
Hepatitis C antibody	Neg	Pos	Pos	Neg	Pos	Neg	Pos	Pos	Pos	Pos
Hepatitis C RNA	Neg	Neg	Neg	Neg	Neg	Neg	Neg	Neg	Neg	Neg
Anti-AAV8 IgG antibody (Relative units)	1	12	37	1	5	8	1	6	6	5
***Post gene transfer assessment***										
***Steady state FIX activity*** ***IU/dL±SD)^d^***	1.9±0.7	1.5±0.7	2.7±1.3	1.9±0.8	3.0±1.4	5.8±2.0	4.9±0.7	7.0±1.0	5.5±1.3	2.7±1.9
***Elevation of ALT*** ***between weeks 1 and 12*** ***post infusion)***	No	No	No	No	Yes (week 7)	Yes (week 9)	Yes (week 8)	No	No	Yes (week 9)
***Elevation of ALT*** ***between week 13 and 10*** ***years post infusion***	No	No	No	No	No	No	No	No	No	No
***FIX inhibitor***	Neg	Neg	Neg	Neg	Neg	Neg	Neg	Neg	Neg	Neg
***Follow-up period (years)***	13.75	13.42	13.25	13.17	13	12.92	11.83	11.58	11.33	11.08
***Annual usage of FIX concentrate***										
Pre-gene transfer (IU/Kg)	3608^a^	4284 ^a^	2486 ^a^	1515 ^a^	2678 ^a^	2509 ^a^	5719 ^b^	3130 ^a^	1367 ^b^	1714^b^
Post gene transfer (IU/Kg)	798	3559	2723	17	1863	35	497	237	45	106
***Annual bleeding rate***										
Prior to gene transfer	3^a^	13^a^	12^a^	12^a^	15^c^	3.3 ^c^	22^b^	20^b^	36^b^	29^b^
Mean ABR post gene transfer	1.9	4.6	1.9	0.7	5.5	0.3	2.3	0.9	1.1	0.2

vg = vector genome copies, kg = kilogram, FIX = factor IX, CRM = cross-reacting material (CRM+ patients have near normal levels (at least 30%) of dysfunctional protein in their plasma. FIX antigen is not detectable in CRM-patients.), HIV = human immunodeficiency virus, AAV8 = adeno-associated viral vector subtype 8, IgG = immunoglobulin G, SD = standard deviation, ALT = alanine aminotransferase, ± = plus or minus.

a Average of two years

b one year

c three years of data.

d Steady state FIX activity and antigen levels were defined as the mean of all levels obtained from 4 months after gene transfer through the December 31, 2023, and where, in each case, the most recent dose of recombinant FIX protein was at least ten days prior.
