# Supplementary material for: Sustained clinical benefit of AAV gene therapy in severe hemophilia B
Source: N Engl J Med. Author manuscript; Available in PMC 2025 Jul 4. (PMC7617823; doi:10.1056/NEJMoa2414783)
Supplement: Supplement [file EMS204449-supplement-Supplement.pdf]

Supplementary Appendix

This appendix has been provided by the authors to give readers additional information about the authors' work.

Supplement to: **Ulrike M. Reiss, Andrew M. Davidoff, Edward G.D. Tuddenham, et al. Sustained clinical benefit of AAV gene therapy in severe hemophilia B**

Contents

List of Members of the Trial Management Group	Page 2
Supplementary Methods	Page 3
Statistical analysis	Page 9
Supplementary Results	Page 10
Immunosuppression regimens	Page 10
Compliance with annual follow-up	Page 10
Clinical impact of gene therapy by annualized bleed rate and factor IX concentrate use over time	Page 10
Details on cancer-related SAEs	Page 11
Figure S1: Annualized bleed rate across time	Page 13
Figure S2: Factor IX concentrate use over time	Page 14
Figure S3: Liver biopsy analysis from subject 8	Page 15
Figure S4: Total IgG AAV8 antibody titer before and after gene therapy with scAAV2/8-LP1-hFIXco	Page 16
Figure S5: In-vivo transduction inhibition assay	Page 17
Table S1: Protocol synopsis	Page 18
Table S2: Summary of adverse events by vector dose level	Page 21
References	Page 22

List of Members of the Trial Management Group

Amit C. Nathwani, Chief Investigator, Royal Free Hospital

Ulrike M. Reiss, Principal Investigator, St. Jude Children's Research Hospital

Edward G.D. Tuddenham, Co-Investigator, Royal Free Hospital

Andrew M. Davidoff, Co-Investigator, St. Jude Children's Research Hospital

Allison Evans, Regulatory Manager, UCL Joint Research Office

Gail Fortner, Project Manager, St. Jude Children's Research Hospital

Pratima Chowdary, Co-Investigator, Royal Free Hospital

Arnulfo J. Pie, Study Nurse, Royal Free Hospital

Supplementary Methods

Vector description. As described before, the scAAV-LP1-hFIXco expression cassette includes an intact 5' terminal resolution site (trs), a deleted 3' trs, and a liver-specific (LP1) enhancer/promoter. It is situated upstream of a modified SV40 small t antigen intron, followed by a codon-optimized hFIX cDNA and an SV40 poly A sequence.[1,2]

Vector manufacturing. Two scAAV2/8-LP1-hFIXco vector lots were produced in accordance with cGMP standards by Children's GMP, LLC, a St. Jude Children's Research Hospital-owned company. The manufacturing process involved transient transfection of adherent human embryonic kidney cells with an adenoviral helper plasmid and a chimeric AAV2 Rep-8Cap packaging plasmid to generate AAV8-pseudotyped vector particles. These particles were purified using ion exchange chromatography. Vector titer was determined using a gel-based assay.[3] Full, assembled AAV8 capsids were assessed using the AAV8 Titration ELISA Kit (PRAAV8, Progen Biotechnik GmbH, Heidelberg, Germany). The table below summarizes the empty to full ratio as well as other critical parameters.

Parameters	Lot 1: 08-055	Lot 2: 09-001
Titer	2.36×10^{12} vg/mL	2.03×10^{12} vg/mL
% Empty	89.6	89.3
% Full	10.4	10.7
Participants treated, n (Number)	6 (#1 to #6)	4 (#7 to #10)

Total AAV antibody ELISA. An immunocapture assay was employed to detect specific antibodies against AAV8, AAV5, or AAV3b in human plasma derived from subjects exposed to scAAV2/8-LP1-hFIXco. Briefly, plates were coated with AAV particles at a concentration of 1×10^{10} vector genome copies/mL in phosphate buffered saline (PBS). After washing and blocking, samples and controls in diluent (2% bovine serum

albumin (BSA), 2% negative plasma pool, PBS-T) were incubated for 2 hours. Antibodies against AAV were identified using Protein G HRP (Sigma P8170) diluted 1:50,000 in peroxidase stabilization buffer (Rockland Immunochemicals) for 1 hour at 37°C. Antibody titers were determined from a standard curve prepared with IgG (Sigma 56834) using a 4-parameter curve.

In vivo transduction inhibition assay. Our previously described screening method was adapted to assess the transduction inhibition potential of anti-AAV neutralizing antibodies in human plasma post gene therapy.[4] Briefly, 50 µl of test plasma was injected into male C57Bl/6 mice via tail vein. Mice in the positive control group received plasma from a subject with high-titer neutralizing anti-AAV antibodies (AAV8 +ve plasma), while the negative control group received plasma from trial participants before successful dosing (AAV8 –ve plasma) (Figure S5). A PBS control group received PBS instead of plasma. Two hours post-plasma injection, scAAV2/8-LP1-hFIXco or scAAV2/5 LP1-hFIXco ($2x10^{12}$ vector particles per kilogram of body weight) were administered via tail vein. Plasma samples were collected at 5 days post-vector administration and analyzed for human factor IX antigen expression using ELISA (Asserachrom IX:Ag, Stago) as per the manufacturer's instructions. To account for experimental variations, human factor IX antigen levels in experimental animals were normalized against PBS control values.

Neutralizing antibody assay. HEK 293T cells were seeded at $1x10^5$ cells per well in white 96-well plates one day prior. Plasma samples were heat-inactivated at 50°C for 1 hour and then serially diluted eightfold in assay diluent (2% heat-inactivated negative plasma pool in DMEM). These diluted samples were incubated for 1 hour at 37°C with AAV-scCMV nLUC in triplicates to achieve final MOIs of 1 for AAV3b or 100 for AAV5 and AAV8. After removing media from HEK 293T cells, the sample/AAV mixture was added in triplicate and incubated for 24 hours. The Nano-Glo luciferase system (Promega) was applied following the manufacturer's instructions, and luminescence was measured with a spectrophotometer plate reader. Relative luminescence units (RLU) were normalized to cells treated with AAV only. Dilution titers were determined by interpolating 50% normalized expression from the dilution series plotted on a log-linear

scale using a 4-parameter curve. The lowest evaluated dilution was 1:5, with samples showing expression greater than 50% at this dilution assigned a dilution titer of 5.

FIX:C measurement. Up to 2015, patient samples were analyzed using a standard one stage factor IX assay on an ACL3000 (Instrumentation Laboratory, Bedford, USA). Briefly, patient samples were diluted 1:5, 1:10, 1:20 in Owrens buffered saline (OBS) and compared to a plasma laboratory standard calibrated against the 3rd international plasma standard (99/826) (NIBSC, Potters Bar, UK) that was diluted 1:5, 1:10, 1:20 and 1:40 in OBS. Patient dilutions were added to lyophilized hereditary factor IX deficient plasma (Technoclone, Vienna, Austria) and APTT lyophilized silica reagent (Instrumentation Laboratory, Bedford, USA), incubated 5 minutes then clotted with CaCl2. FIX:C results were reported as percentage of normal and IU/dL.

From 2015 onwards, patient plasma samples were assayed using one-stage APTT based factor IX assay on an ACL TOP 700 (Werfen/Instrumentation Laboratory (IL), Bedford, USA). Briefly patient samples were diluted in HemosIL factor diluent (Werfen/IL) 1:10, 1:20, 1:40 and compared to an 8-point calibration curve using a traceable calibrator (CryoCheck Normal Reference, Precision BioLogic Inc. Dartmouth, NS, Canada). Patient and calibrator dilutions were added to HemosIL factor IX deficient plasma (Werfen/IL) and HemosSIL APTT SynthASil reagent (Werfen/IL), incubated 3 minutes then clotted with HemosIL CaCl2 (Werfen/IL). Factor IX levels were reported as percentage of normal and IU/dL.

A pairwise comparison of samples by the two different one stage methods optimized for the respective instrumentation showed comparable factor IX activity with low intra-assay variability for controls, 36 patient samples (r^2=0.9755) and 35 samples from gene therapy participants (r^2=0.8596).

Proviral scAAV-LP1-hFIXco viral DNA detection in pathological specimens. Due to the availability of only formalin-fixed, paraffin-embedded (FFPE) tissue, the analysis was limited to a droplet digital PCR (ddPCR) assay for proviral vector DNA. DNA was extracted from FFPE tissues using the Maxwell® RSC DNA FFPE kit (AS1450, Promega, Madison, WI, USA) based on the manufacturer’s instructions, and quantified using

Qubit™ 1X dsDNA HS Assay Kit (Q33230, Thermo Fisher Scientific, Waltham, MA, USA). The QX200™ Droplet Digital™ PCR System (Bio-Rad, Pleasanton, CA) was used for digital PCR. The ddPCR reaction mixture consisted of 10 µL of 2 x ddPCR Supermix (Bio-Rad), 0.4 µmol each of the primers and probe for target, 1 µL of 20 x RNase P primer/probe mix (Thermo Fisher Scientific), and 5 µL of nucleic acid extract in a final volume of 20 µL. The entire reaction mixture was used to produce the droplets on the QX200 AutoDG Droplet Digital PCR System (Bio-Rad). After processing, droplets collected in a 96-well PCR plate (Eppendorf, Germany) were amplified on a C1000 Thermal Cycler (Bio-Rad) beginning at 95°C for 10 minutes, followed by 40 cycles of 94°C for 30 seconds and 60°C for 60 seconds, and 1 cycle of 98°C for 10 minutes, ending at 12°C. The plate was read on the Droplet Reader (Bio-Rad) at a rate of 32 wells per hour. ddPCR data was analyzed with QuantaSoft analysis software (Bio-Rad).

Two sets of TaqMan primer/probes that are specific to the DNA sequence of codon-optimized human factor IX (hFIXco) open reading frame (ORF) and not predicted to bind to the normal human DNA sequence of factor IX (assay one: primers 5'GGCTACCTGCTGTCTGCTGAGT and 5' TTGTATCTCTTGGGCCTGTTCA, probe 5'CCTGGACCATGAGAATGCCAACAAGATC; assay two: primers 5'CCACTGAGTTCTGGAAGCAGTATG and 5' GCTGTTGATGTCATCCTTGCA, and probe 5'FAMCCAGTGTGAGAGCAACCCCTGCCT) were selected using Primer Express software 3.0.1 (Life Technologies, Thermo Fisher Scientific Inc., Carlsbad, CA). The assays were designed to detect DNA sequences of 61-143nt and 251-342nt specific to the hFIXco ORF, respectively. Human genomic DNA was quantified based on detection of the human RNase P gene (a single-copy gene encoding the RNA moiety for the RNase P enzyme) using TaqMan™ RNase P Control Reagents Kit (Thermo Fisher Scientific Inc, Carlsbad, CA) according to the manufacturer's recommendations.

Assay performance characteristics were validated using serial dilutions of known concentrations of LP1-hFIXco vector DNA. Quantitative standards, consisting of ten-fold serial dilutions from $5x10^{-1}$ to $5x10^{5}$ vector genome copies per microliter, were created from the hFIXco vector DNA. The lower limits of

detection (LOD) were determined to be 6.40 and 4.26 vector genome copies of hFIXco per PCR reaction, or 0.003 and 0.002 vector genome copies per human RNase P gene, when a constant amount of human genomic DNA was used in multiplex ddPCR analysis, for assay one and assay two, while analytical detection range was seen from 5-50000 vector genome copies of hFIXco per reaction for both assays. Specificity was then challenged using naïve human genomic DNA. The results showed that the assays were specific to the hFIXco vector DNA, with no cross-reactivity to naïve human DNA. Quantitative standards of 10-fold serially diluted human genomic DNA from $4.2x10^1$ to $4.2x10^4$ copies/µL were created using naïve human genomic DNA and used to determine the detection range and LODs of the assays. A single copy human RNase P gene was targeted to calculate human genomic copy number. The LOD for the RNase P gene was determined to be 26.2 and 11.7 copies/PCR reaction for assays one and two, while analytical detection range from 4.2×10^1 to 4.2×10^4 copies/PCR reaction was determined for both assays.

Quantification of FIXco copy number from patient DNA samples: A total of three groups of de-identified DNA samples were tested using both assays. The results were expressed as the vector copy number per human RNase P gene. Both primer/probe sets were used for each specimen; the results were then reported as an average. This analysis was performed in duplicate (using the same DNA extract) and then reported as an overall mean.

Participant-specific resected tissue was analyzed by a team at St. Jude Children's Research Hospital, separate from the hemophilia gene therapy investigators, once they had been deidentified. Controls were prepared and analyzed in the same way as the pathological tissue and consisted of:

A. DNA from normal human tissue (e.g. negative control consisting of prostate for participant 2 and lung for participant 9) (both the patient's tissue and a separate control human sample) was prepared in the same manner after FFPE tissue.

B. Tissue biopsies of experimental NHPs that had previously been exposed to scAAV2/8-LP1-hFIXco vector (positive control).

Lung screening by CT scans. Low-dose computed tomography (LDCT) was used for lung cancer screening on a 5-yearly basis following the incidental finding of atypical adenomatous hyperplasia (AAH) in a small number of older rhesus macaques exposed to non-clinical grade AAV-FIX vector during preclinical studies. Many of these animals were exposed to histone deacetylase inhibitors and other oncogenic therapies. Molecular studies of the AAH lesions revealed inconsistent results, with no clear association between vector genomes and lesion development. Such lesions were also found in a small number of animals not exposed to AAV vectors.

ISH and RISH method. The RNAscope Multiplex Fluorescent Detection v2 kit (Advanced Cell Diagnostics) was utilised for fluorescent in situ hybridisation to visualise specific DNA and RNA sequences in formalin-fixed, paraffin-embedded liver sections. Custom probes targeting the vector sequence (scAAV2/8-LP1-hFIXco) enabled precise detection of viral DNA/RNA. Hepatocyte membranes were stained with a hepatocyte-specific conjugated HepPar-1 antibody (Novus Biologicals). Normal liver tissue, provided by the Royal Free Hospital Histopathology Department, was used as a control and tested with the same custom FIX probes as the patient samples. Negative and positive control probes from Advanced Cell Diagnostics were also included. Negative probes targeted the DapB gene (accession #EF191515) from Bacillus subtilis strain SMY, while positive probes targeted housekeeping genes, specifically RNA polymerase II subunit RPB1 and cyclophilin B. Imaging was conducted using a Zeiss LSM900 confocal microscope with Plan-Apochromat 20x and 40x objectives, and images were processed using Zeiss Zen microscopy software.

Criteria for identifying a target joint. A target joint was defined as per the consensus definition established by the Factor VIII, Factor IX and Rare Coagulation Disorders Subcommittee of the Scientific and

Standardization Committee (SSC) of the International Society of Thrombosis and Haemostasis (ISTH), by three or more spontaneous bleeds into a single joint within a consecutive 6-month period.[5] Where there have been ≤2 bleeds into the joint within a consecutive 12-month period the joint is no longer considered a target joint.

Statistical Analysis

Efficacy, a secondary endpoint, was evaluated through factor IX activity, annualized bleeding rate (ABR), and factor IX concentrate usage, summarized for pre-gene therapy and as of December 31, 2023. A linear mixed-effects model assessed the impact of dosing (high vs. low/intermediate) on factor IX activity and the influence of time post-therapy on this relationship. Steady-state factor IX activity was determined by averaging the accumulation of all factor IX activity after month 4 post-infusion (once transaminitis resolved and corticosteroid treatment ceased). Low and intermediate dose groups were combined for model analysis and comparisons. For all factor IX activity-related analyses, only factor IX levels measured at least 10 days after factor IX use were included. Pre-gene therapy ABRs and factor IX use were based on prior reports.[2,3] Efficacy was measured as fold change and reported using medians and interquartile ranges [IQR]. For the fold change between participants and external controls, the observed ratio of medians is reported along with the medians and IQR of both groups. Analyses were conducted using SAS 9.4 software (SAS Institute) and R version 4.3.2[6] packages tidyverse[7] for figures.

Supplementary Results

Immunosuppression regimens used in participants with vector-induced transaminitis. Four participants (#5, #6, #7, and #10) who were treated with the high vector dose of $2x10^{12}$ vg per kilogram of body weight developed a single episode of asymptomatic vector-induced elevation of alanine aminotransferase (ALT) with peak levels between 36 and 202 IU per liter. These individuals initiated prednisolone treatment at 60

mg orally daily between weeks 7-10, and tapered over a median of 8 weeks (range: 8-12 weeks). Elevated ALT levels resolved within a median of 5 days (range: 2-35 days). Factor IX activity levels declined by a median of 52% (range: 0-70%) from peak values preceding ALT elevation.

Compliance with annual follow-up

During long-term follow-up, we quantified compliance as the proportion of protocol-mandated annual visits attended. Seven participants attended 100% of visits, one attended 83%, and two attended 40% and 50%, respectively.

Clinical impact of gene therapy by annualized bleed rate and factor concentrate use over time

Long term clinical impact of scAAV2/8LP1-hFIXco was assessed across the duration of the follow-up period by assessing annualized bleed rates (Figure S1) and annualized use of factor IX replacement therapy (Figure S2), (Post-GT) compared with a minimum of 12 months prior to gene therapy (Pre-GT). As shown in Figure S1 there was a sustained 9.7-fold (IQR, 3.7 to 21.8) reduction in bleeding events compared to the pre-treatment period. In the 6 high-dose participants, the drop in ABR was a median of 16.4-fold (IQR, 9.65 to 31.3) lower. Figure S2 shows that factor IX concentrate usage decreased by a median of 12.4-fold (IQR, 2.21 to 27.1) over 13 years post-gene therapy in all 10 participants, reaching 14.7-fold (IQR, 11.9 to 27.1) in the high dose cohort.

Further details of cancer-related SAEs

Participant 2: At 74 years old, and 11.6 years after receiving the low dose ($2x10^{11}$ vector genome copies per kilogram body weight) of AAV vector, Participant 2 was found to have an elevated prostate-specific antigen (PSA) level of 47 ng/mL. A prostate biopsy revealed high-risk, T2 localized prostate adenocarcinoma with a Gleason score of 4+4=8. The patient subsequently underwent radiotherapy, luteinizing hormone-releasing hormone (LHRH) agonist injections, and treatment with alendronic acid.

Proviral vector DNA was not detected in the adenocarcinoma samples by droplet digital PCR assay as described in the methods section. Positive control DNA extracted from a monkey liver specimen previously transduced with the scAAV2/8 LP1-hFIXco vector using the same method and analysed at the same time showed a copy number of approximately 32 vector DNA per human RNase P gene as expected. No vector DNA was detected in a naïve monkey specimen or in commercial human DNA. This adverse event was reported to regulatory authorities as serious and possibly related to the investigational product. However, it likely was not associated with the gene therapy, given the common prevalence of prostate adenocarcinoma in individuals of this participant's age group.

Participant 8: A life-long smoker (10-15 cigarettes per day, approximately 10 pack-years, from the age of 17 years) received the scAAV2/8-LP1-hFIXco vector at the high dose of $2x10^{12}$ vector genome copies per kilogram body weight. Seven years after gene therapy, at age 44 years, he was diagnosed with a 10-mm non-mucinous adenocarcinoma in situ in the left upper lobe of the lung, discovered incidentally following a bullectomy and left talc pleurodesis for a spontaneous pneumothorax. The lesion was surgically removed, and he has not required further treatment. He remains clinically well with no recurrence on annual CT scans.

Small, formalin-fixed paraffin-embedded lung tissue blocks containing either normal tissue or normal tissue with admixed pathological lung tissue were analyzed at St. Jude Children's Research Hospital by an independent team after de-identification. Using standard histologic (hematoxylin & eosin) preparations as a guide, pathologic tissue was macrodissected from unstained sections on slides under sterile conditions and DNA extracted from this tissue prior to droplet digital PCR analysis. A small amount of scAAV-LP1-hFIXco vector DNA was detected in both the carcinoma in situ and normal surrounding lung tissue; the number of copies detected in pathologic lung was similar to that detected in surrounding normal lung tissue (0.04 vector genome copies per RNaseP gene vs 0.05 vector genome copies per RNaseP gene, respectively). The root cause of the adenocarcinoma remains unknown due to insufficient tumor tissue

for further testing. This adverse event was reported to regulatory authorities as serious and possibly related to the investigational product but may have been an incidental occurrence in an individual with an extensive smoking history.

Figure S1: Annualized bleed rate (ABR, median and interquartile range (IQR) of bleeds per year) across time in all participants (top panel), low and intermediate-dose participants combined (lower doses, middle panel), and high-dose participants (bottom panel). Box-plots include l ABR median (horizontal bar inside box), interquartile range (top bar [Q1=25th percentile] and bottom bar [Q3=75th percentile]), and whiskers (T bars extending from Q1 to the smallest value up to the Q1-1.5xIQR and from Q3 to the largest value up to Q3+1.5xIQR). Dots represent participant ABR. Dots above or below the boxplot are participant ABR that are less than the 25th percentile+(1.5xIQR) or more than the 75th percentile-(1.5xIQR). ABRs for Participant 5 in the high dose and Participant 2 in the low dose group were outliers post gene therapy infusion.

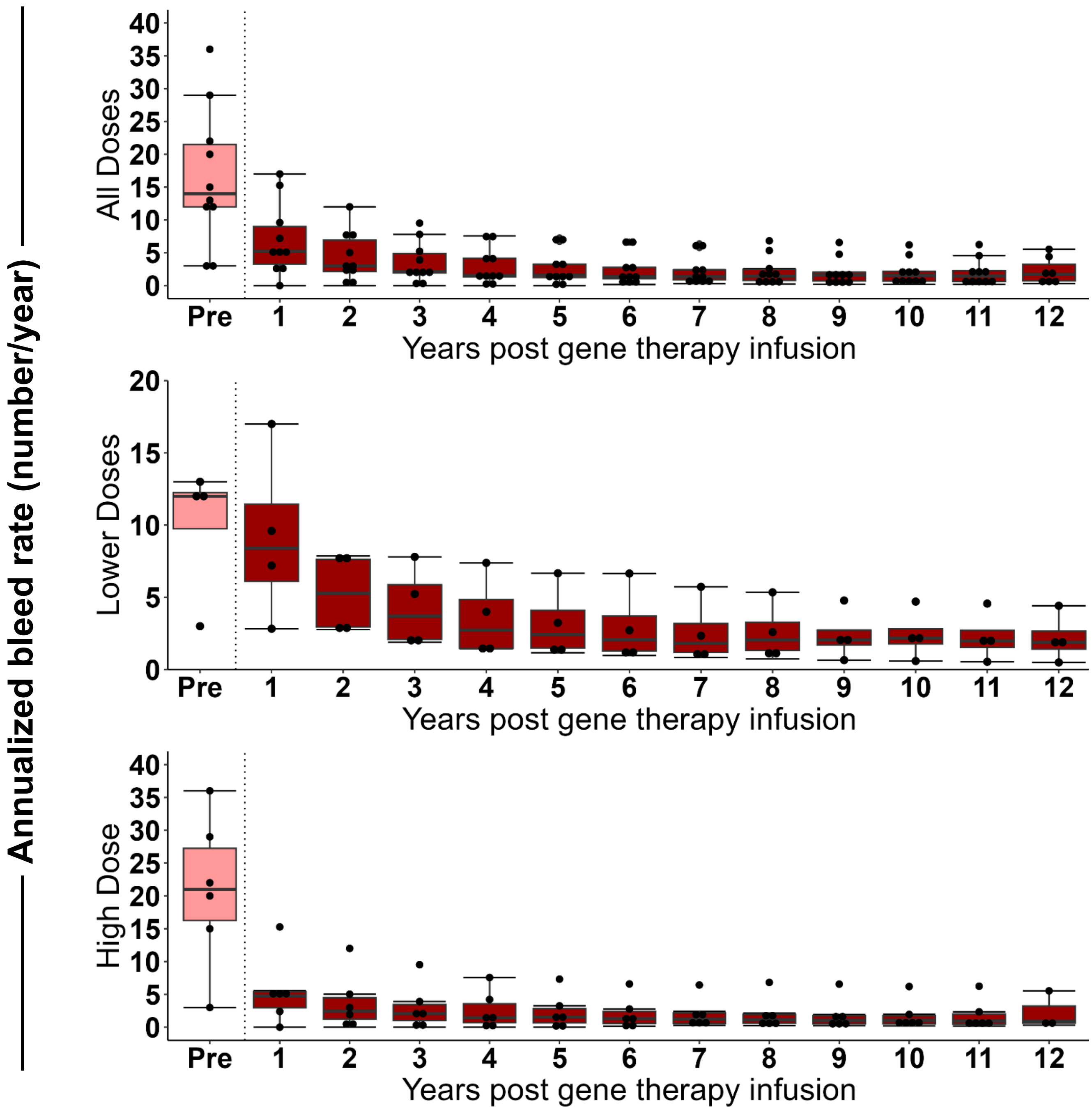

Figure S2. Factor IX use (median and interquartile range (IQR) of annual factor IX use) across time for all participants (top), low and intermediate-dose participants combined (middle), and high-dose participants (bottom). Box-plots include median (horizontal bar inside the box), interquartile range (top bar [Q1=25th percentile] and bottom bar [Q3=75th percentile]), and whiskers (T bars extending from Q1 to the smallest value up to the Q1-1.5xIQR and from Q3 to the largest value up to Q3+1.5xIQR) for annual factor IX use. Dots represent participant's factor IX use averaged over the year. Dots above or below the boxplot are mean factor IX usage that was less than the 25th percentile+(1.5xIQR) or more than the 75th percentile-(1.5xIQR). Factor use for Participant 5 in the high dose and Participant 2 in the low dose group were outliers post gene therapy infusion.

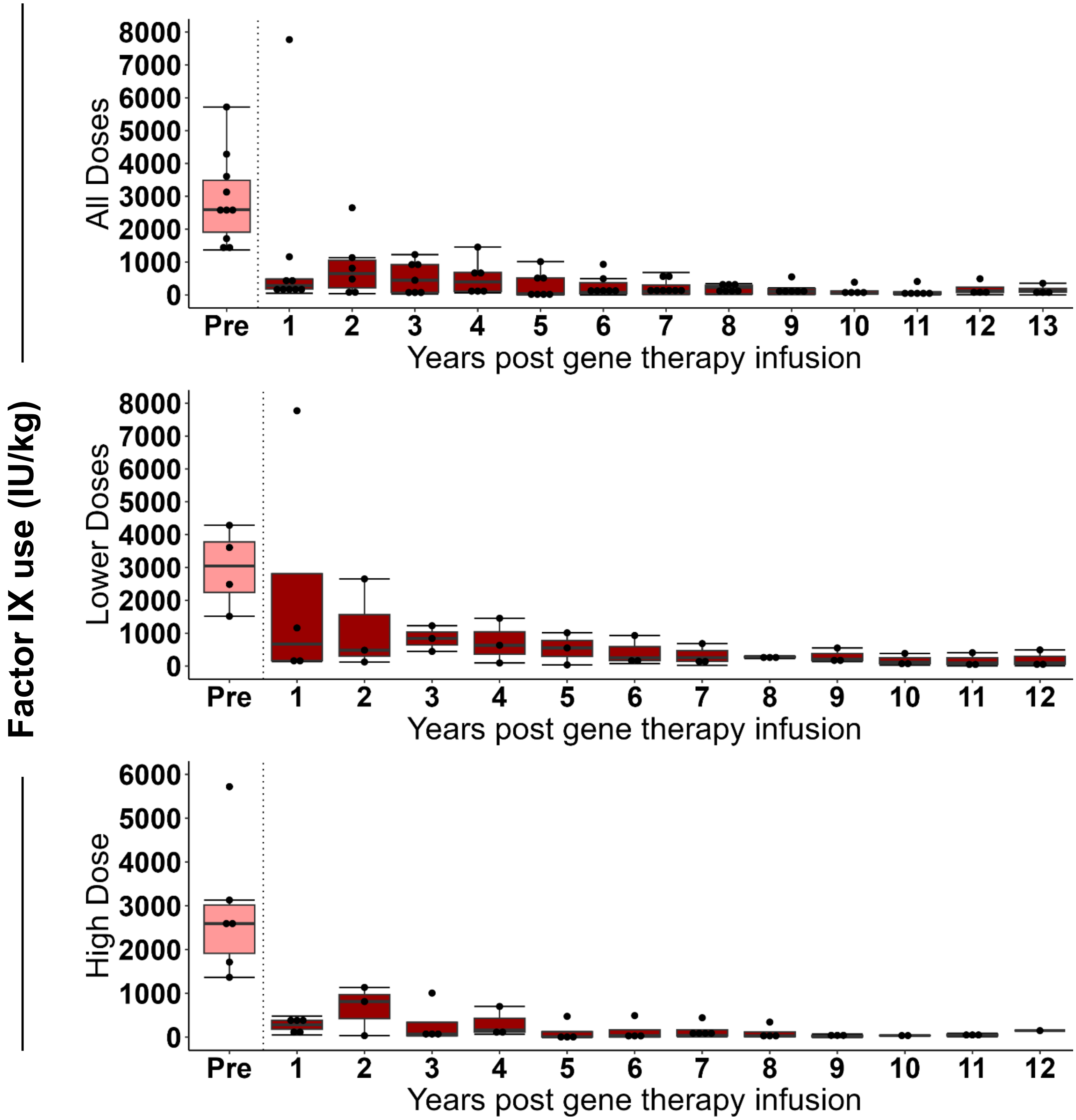

Figure S3: Liver biopsy analysis from subject 8 at 10 years post-gene transfer. A. Representative biopsy sections from subject 8 treated with scAAV2/8-LP1-hFIXco at a dose of $2x10^{12}$vg per kilogram. **B.** H&E staining shows preserved lobular architecture and hepatic plates with no hepatocellular injury. Asterisk indicates a portal tract, and arrow marks a hepatic venule (200x magnification). **C.** In-situ hybridization using codon-optimized FIX probes show transgenic FIX DNA (red dots) in hepatocyte nuclei, identified by HepPar1 immunofluorescence (purple). scale bar is 500 µm. Cytoplasmic FIX transcript (green) is detected by FISH in some of the hepatocytes. Each red or green dot represents at least one vector genome/transcript, with possible multiple copies in a single focus. Magnified area shows transduced hepatocytes (white arrow) with or without transcriptional activity.

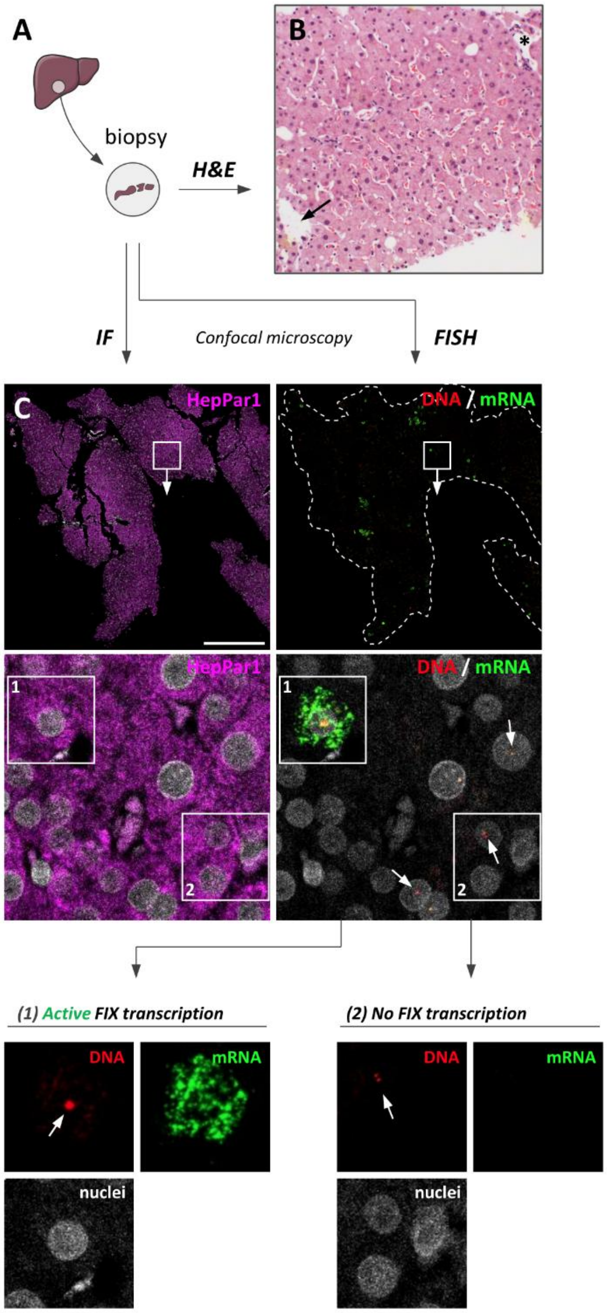

Figure S4: Total IgG AAV8 antibody titer before and after gene therapy with scAAV2/8-LP1-hFIXco. (A) Total IgG AAV8 antibody levels (TAB, median±Interquartile range) by ELISA in serum samples collected at various time points following gene therapy with scAAV2/8-LP1-hFIXco compared to pre-therapy levels in the same individuals and to levels in a group of normal controls (NC). **(B)** Total IgG AAV8 antibody levels (TAB, median±Interquartile range) in humans and rhesus macaques positive for antibodies following infection with wild type AAV8 (clear bars) or after exposure to a similar dose of the scAAV2/8-LP1-hFIXco vector (grey bars) at 3 months post-gene therapy.

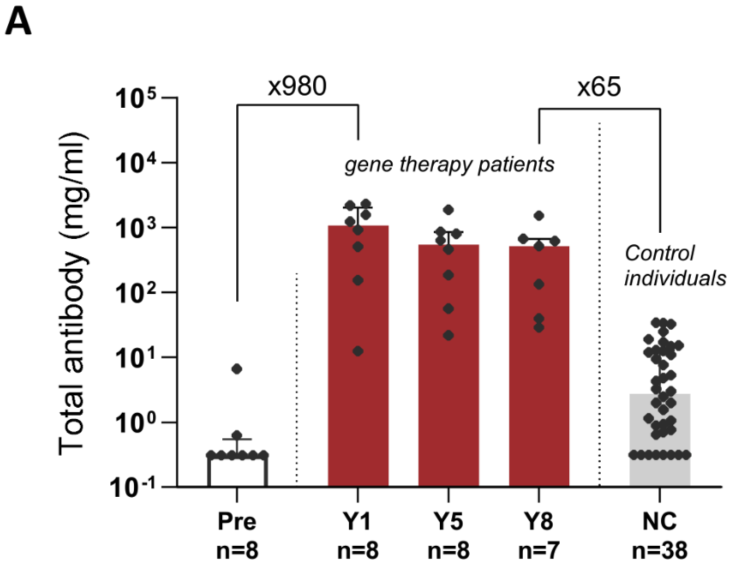

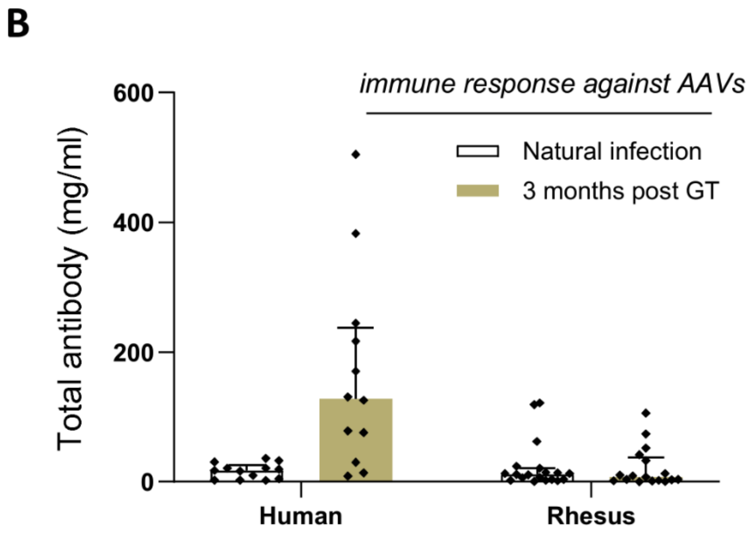

Figure S5: In-vivo transduction inhibition assay. Serum from human subjects, collected 8-10 years post-administration of low-dose (Subject 1) or high-dose (Subject 5 and 6) scAAV2/8-LP1-hFIXco, was passively transferred to C57BL/6 mice (n=3 per cohort). These mice subsequently received a single dose of scAAV-LP1-hFIX pseudotyped with **(A)** AAV8 or **(B)** AAV5 capsids. The transduction inhibition potential of the human serum was assessed by measuring human factor IX expression in the mice 5 days post-gene transfer. AAV-positive and AAV-seronegative human plasma served as positive and negative controls, respectively. Data are presented as median±Interquartile range).

A

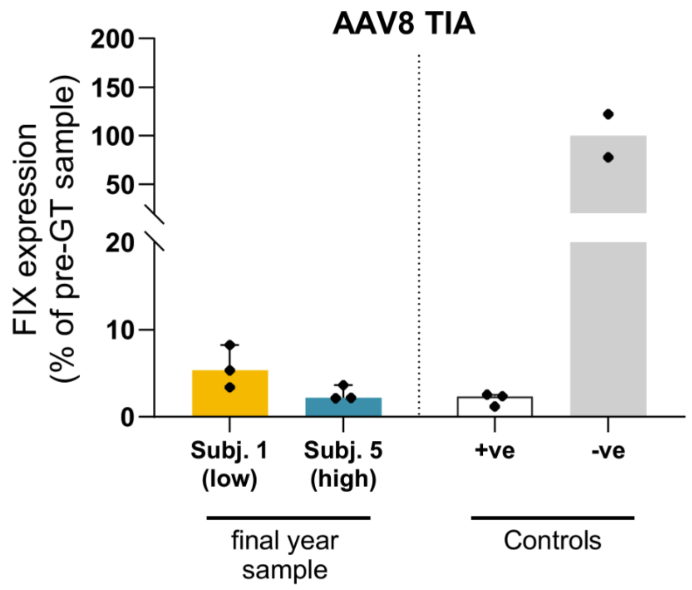

B

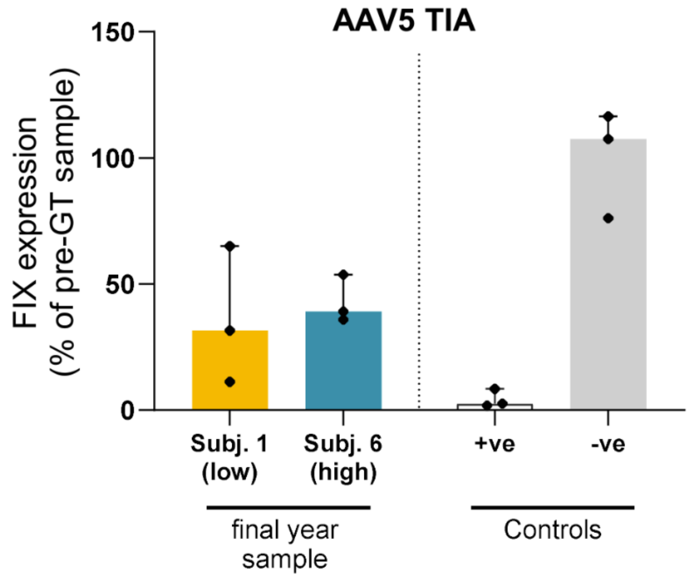

Table S1: Protocol synopsis

**Study title**	An open label dose-escalation study of a self-complementary adeno-associated viral vector (scAAV2/8-LP1-hFIXco) for gene transfer in subjects with severe hemophilia B
**Clinicaltrials.gov Identifier:**	NCT00979238
**Study Centers:**	(1) Katharine Dormandy Haemophilia Centre and Thrombosis Unit (KDHCTU) which is part of the University College London, London, UK<br>(2) St Jude Children's Research Hospital, Memphis, TN, USA
**Objectives**	
**Primary Objective:** Assess the safety of systemic administration of a novel self- complementary AAV vector (scAAV2/8-LP1-hFIXco) in adults with severe hemophilia B at three different dose levels consisting of 2×10^{11}, 6×10^{11} and 2×10^{11} vector genomes per kilogram (vg/kg) of body weight as titered by a gel-based method.<br><br>**Secondary Objective:**<br>a. Estimate the dose of scAAV required to achieve stable expression of hFIX at or above 3% of normal (≥ 3u/dl).<br>b. Establish the kinetics, duration and magnitude of scAAV-mediated hFIX expression in individuals with hemophilia B for a given vector dose.<br>c. Describe the immune responses to the hFIX transgene product and AAV capsid proteins following systemic administration of scAAV2/8-LP1-hFIXco.<br>d. Assess viral shedding in various body fluids after systemic administration of scAAV2/8-LP1-hFIXco.	
**Study Design**	
An open label dose-escalation, Phase I/II study entailing peripheral vein administration of a single dose of a novel self-complementary AAV (scAAV2/8-LP1-hFIXco) vector into adult subjects with severe HB following informed consent. Dosing will begin with a low dose (2×10^{11} vg/kg) and progress to an intermediate dose level (6×10^{11} vg/kg) and then to the highest dose of 2×10^{12} vg/kg. Between 2-6 patients may be enrolled at each of 3 dose levels depending on toxicity and FIX levels. However, only one subject will be treated at a time and observed for at least 42 days prior to the enrolment of the next subject. The dose level will be based on a gel titration method which allows direct visualization and quantitation of the titer by comparison of intensity of bands from the vector to a series of standards. A comprehensive monitoring schedule has been established to assess the primary end point of safety which includes an array of clinical and laboratory evaluations including liver biochemistry, semen analysis for vector genomes, and immunological response to hFIX and AAV capsid. Accrual will be suspended if dose limiting toxicity, including any Grade III-IV adverse events or any Grade II adverse events that persist for more than 7 days and are at least possibly related to the vector product occur in any of the enrolled subjects.	
**Main Eligibility Criteria**	

**Inclusion criteria:**	1.	Males ≥ 18 years of age with established severe HB (FIX:C<1u/dl) resulting from a mutation in the hFIX gene which has not been associated with an inhibitor in the database (http://www.biochem.ucl.ac.uk/pavithra/fix/structure.html.php) with detectable FIX in serum.
	2.	Treated/exposed to FIX concentrates for at least 10 years,
	3.	A minimum of an average of 3 bleeding episodes per year requiring FIX infusions or prophylactic FIX infusions because of frequent prior bleeding episodes,
	4.	Able to give informed consent and comply with requirements of the trial,
	5.	Currently free of inhibitor and have no history of inhibitors to FIX protein, and
	6.	A negative family history for the development of an inhibitor,
	7.	Willing to practice a reliable barrier method of contraception.
**Exclusion criteria:**	1.	Evidence of active infection with Hepatitis B or C virus as reflected by HBsAg or HCV RNA positivity, respectively. To be considered negative for active infection, two negative assays at a minimum of a six-month interval were required,
	2.	Exposure to Hepatitis B or C and on antiviral therapy,
	3.	Serological evidence of HIV or HTLV infection,
	4.	Significant liver dysfunction as defined by an abnormal ALT (alanine transaminase), bilirubin, alkaline phosphatase or INR. Potential participants who have had a liver biopsy in the past 3 years were excluded if they had significant fibrosis of 3 or 4 as rated on a scale of 0-4,
	5.	Coronary artery disease as a co-morbid condition,
	6.	Platelet count of $<150 \times 10^9$/l,
	7.	Creatinine ≥ 1.5 mg/dl,
	8.	Hypertension with systolic BP consistently ≥ 130mmHg or diastolic BP consistently ≥ 90mmHg,
	9.	History of active tuberculosis, fungal disease or other chronic infection,
	10.	History of chronic disease adversely affecting performance,
	11.	Detectable antibodies reactive with AAV8,
	12.	Subjects who were unwilling to provide the required semen samples,
	13.	Poor performance status (WHO performance status score >1), or
	14.	Received an AAV vector previously or any other gene transfer agent in the previous 6 months.

**Study Procedures/ Frequency:**	Following vector administration, subjects will be evaluated at frequent intervals for local or systemic toxicity, efficacy and biodistribution over a period of 1 year followed by regular follow-up for fifteen years.
**Criteria for Evaluation:**	**Primary Endpoint:** Safety, defined as the development of dose-limiting toxicity including any Grade III-IV adverse events or any Grade II adverse events that persist for more than 7 days and are at least possibly related to the study agent, according to the modified symptom specific NCI Common Terminology Criteria for Adverse Events.<br>**Secondary Endpoint:** Efficacy, defined as persistent expression of functional human FIX at > 3% of normal levels in plasma.
**Treatment of transaminitis**	Prednisolone as the sole medication will be used as per the AASLD guidelines with a slight modification:<br>Prednisolone dose (mg/day)<br>Week 1 60<br>Week 2 40<br>Week 3 30<br>Week 4 20<br>Maintenance until ALT returns to base line and then reduce by 5mg/week. More rapid taper was permitted in subjects where ALT had returned to baseline values within week 1 of steroid therapy.

Table S2: Summary of adverse events by vector dose level

	Total Number of Adverse Events	Total adverse events split across dose cohorts					
		2 x 10^{11} vg/kg (n=2)		6 x 10^{11} vg/kg (n=2)		2 x 10^{12} vg/kg (n=6)	
		Incidence	Number of Events	Incidence	Number of Events	Incidence	Number of Events
**Any adverse event**	354	2 (100%)	98	1 (100%)	32	6 (100%)	224
**Serious adverse events**	**11**		**3**		**1**		**6**
Probably related							
Transaminases increased	4					1 (17%)	4
Possibly related							
Adenocarcinoma of the prostate	1	1 (50%)	1				
Non-mucinous adenocarcinoma in situ of the lung	1					1 (17%)	1
Unrelated							
Joint infection	1	1 (50%)	1				
Pneumothorax	1					1 (17%)	1
Sinus bradycardia	1			1 (50%)	1		
Tibia Fracture	1	1 (50%)	1				
**Adverse events related to scAAV2/8-LP1-hFIXco**	**15**		**2**		**1**		**12**
Adenocarcinoma of the prostate	1	1 (50%)	1				
Blood Hematocrit decreased	3					2 (33%)	3
Blood RBC decreased	1					1 (17%)	1
Hypertension	1	1 (50%)	1				
Non-mucinous adenocarcinoma in situ of the lung	1					1 (17%)	1
Splenomegaly	1			1 (50%)	1		
Transaminases increased	7					2 (33%)	7

References

1. Nathwani AC, Gray JT, Ng CY, et al. Self complementary adeno-associated virus vectors containing a novel liver-specific human factor IX expression cassette enable highly efficient transduction of murine and nonhuman primate liver. Blood 2006;107:2653-2661.
2. Allay JA, Sleep S, Long S, et al. Good manufacturing practice production of self-complementary serotype 8 adeno-associated viral vector for a hemophilia B clinical trial. Hum Gene Ther 2011;22(5):595-604.
3. Fagone P, Wright JF, Nathwani AC, et al. Systemic errors in quantitative polymerase chain reaction titration of self-complementary adeno-associated viral vectors and improved alternative methods. Hum Gene Ther Methods 2012;23:1-7.
4. Nathwani AC, Tuddenham EG, Rangarajan S, et al. Adenovirus-associated virus vector-mediated gene transfer in hemophilia B. N Engl J Med 2011;365:2357-65.
5. Blanchette VS, Key NS, Ljung LR, et al. Definitions in hemophilia: communication from the SSC of the ISTH. J Thromb Haemost 2014;12(11):1935-1939.
6. R Core Team. R: A language and environment for statistical computing. R Foundation for Statistical Computing, Vienna, Austria. https://www.R-project.org/. 2023.
7. Wickham H, Averick M, Bryan J, et al. Welcome to the tidyverse. J Open Source Softw 2019;4(43):1686, doi.org/10.21105/joss.01686.
